# The regulatory role of *ZFAS1/*miRNAs/mRNAs axis in cancer: a systematic review

**DOI:** 10.32604/or.2024.050548

**Published:** 2025-02-28

**Authors:** RAHUL KUMAR SINGH, SUROJIT MANDAL, ADRIJA MOHANTA, RITU YADAV, RAJIV RANJAN KUMAR, RINKU KHATKAR, VIVEK UTTAM, UTTAM SHARMA, MANJIT KAUR RANA, MANJU JAIN, HARDEEP SINGH TULI, AKLANK JAIN

**Affiliations:** 1 Non-Coding RNA and Cancer Biology Laboratory, Department of Zoology, Central University of Punjab, Ghudda, Bathinda, 151401, India; 2 Department of Pathology/Lab Medicine, All India Institute of Medical Sciences, Bathinda, 151001, India; 3 Department of Biochemistry, Central University of Punjab, Ghudda, Bathinda, 151401, India; 4 Department of Biotechnology, Maharishi Markandeshwar (Deemed to be University), Mullana, Ambala, 133207, India

**Keywords:** LncRNA, miRNA, mRNAs, Zinc finger antisense 1 (*ZFAS1*), Cancer biomarker

## Abstract

**Objectives:**

Recently, we and others have demonstrated the involvement of Zinc Finger Antisense 1 (*ZFAS1*) in cancer development. However, the intricate interplay of *ZFAS1* with miRNAs and mRNAs remains to be fully understood.

**Materials and methods:**

We followed PRISMA guidelines to retrieve and assess the available literature on the topic “*ZFAS1*/miRNA/mRNA axis” and “Cancer” from databases such as PubMed, Google Scholar, and ScienceDirect. We also used bioinformatic webtools for analyzing the potential miRNA targets of *ZFAS1* and its role in survival of cancer patients along with their role in various biological functions and pathways.

**Results:**

Our literature search and bioinformatic analysis reveals that *ZFAS1* serves as a sponge for numerous miRNAs. Among the various targeted miRNAs, miR-150-5p stands out as significantly correlated with *ZFAS1* across multiple databases (*p*-value = 3.27e−16, *R-*value = −0.346). Additionally, our Kaplan-Meier survival analysis indicates a noteworthy association between *ZFAS1* expression levels and overall poor prognosis and survival rates in ovarian, sarcoma, and pancreatic cancers. We also underscore the involvement of various signaling pathways, including Signal Transducer and Activator of Transcription 3 (STAT3), Spindle and Kinetochore-associated Protein 1 (SKA1), Lysophosphatidic acid receptor 1 (LPAR1), and Wnt β-catenin, in cancer development through the *ZFAS1*/miRNAs/mRNAs axis. Furthermore, we identify *ZFAS1*’s pivotal roles in diverse molecular processes, such as RNA binding and ribonucleoprotein formation.

**Conclusion:**

In conclusion, this review comprehensively summarizes the latest advancements in understanding the regulatory relationships among *ZFAS1*, miRNAs, and mRNAs, emphasizing their collective role in cancer development to propose innovative avenues for cancer treatment. We believe that the intricate relationship among the *ZFAS1*-miRNA-mRNA axis may yield potential therapeutic targets for effective cancer management.

## Introduction

The unregulated expansion and spread of aberrant cells within the body is a hallmark of cancers. These aberrant cells can transform into tumors, infiltrate surrounding tissues, and spread using the lymphatic or circulatory pathways to other areas of the body. The precise origins of cancer are frequently multifaceted, encompassing a confluence of genetic, environmental, and lifestyle elements. A person’s genetic predisposition, tobacco use, excessive alcohol consumption, poor diet, inactivity, exposure to carcinogens, and certain infections like HPV are risk factors for developing cancer.

**Figure 1:**
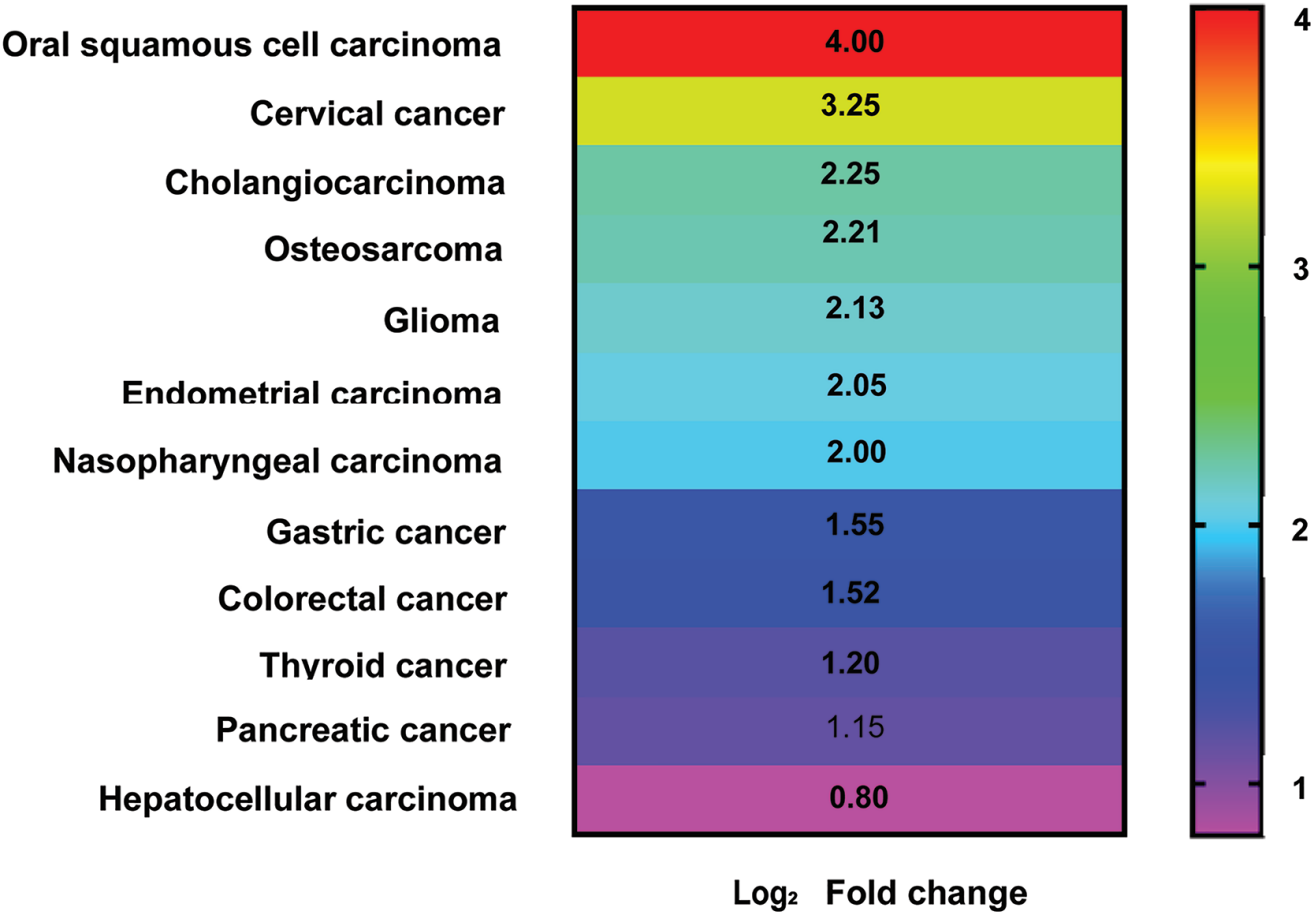
Heatmap showing approximate Log_2_ Fold change in lncRNA *ZFAS1* expression level associated with different cancer types (graph prepared using GraphPad v8.0).

The human genome contains roughly 2% of protein-coding RNA and more than 90% of non-coding RNA [1]. One of the subtypes of these non-coding RNAs is long non-coding RNAs (lncRNAs) having nucleotide lengths between 200–100,000 bases [2]. The half-lives of these lncRNAs range from less than 2 h to more than 16 h, with a median half-life of about 3.5 h [1]. According to the most recent data from LNCipedia (https://lncipedia.org/, version 5.2, accessed 10 March 2024), there are 127,802 lncRNA transcripts present in the mammalian system. Among them, *ZFAS1*, which is the interest of this article, was first discovered in breast cancer (BC). The most common cancer in women and second in lethality. *ZFAS1* has a gene ID: 441951, It is also known as HSUP1; HSUP2; C20orf199; NCRNA00275, and is present on chromosome 20q13.13 on the antisense strand of zinc finger NFX1-type containing 1 [3]. It has five exons with five transcriptional variations (NR_003604.3:1008 bp; NR_003605.2:689 bp; NR_003606.3:860 bp; NR_036658.2:946 bp; NR_036659.2:504 bp), and its nucleotide length is around 17561 bp [3,4]. Recently, we demonstrated for the first time the significant downregulation of *ZFAS1* in triple-negative breast cancer (TNBC) patients compared to matched healthy control samples. We also reported that *ZFAS1* promotes TNBC pathogenesis by negatively affecting the activity of the STAT3 gene in MD Anderson-Metastatic Breast (MDA-MB-231) cells. We have also shown that low expression of *ZFAS1* leads to increased expression of epithelial-to-mesenchymal transition marker proteins in MDA-MB-231 cells [4]. *ZFAS1* plays a significant role in several other malignancies such as hepatocellular carcinoma [5], oral squamous cell carcinoma [6] cervical carcinoma [7], thyroid cancer [8], cholangiocarcinoma [9], glioma [10], endometrial carcinoma [11], pancreatic cancer [12], osteosarcoma [13], colorectal cancer [14], gastric cancer [15], and nasopharyngeal carcinoma [16] as shown in Fig. 1.

Target mimicry is the mechanism by which lncRNAs with endogenous target mimic (eTM) sites interact with miRNAs (nucleotide length 18–24). miRNAs are seized by lncRNAs through complementary binding, limiting their effects. This is referred to as decoying or sponging [17]. This lncRNA-miRNA crosstalk, based on the miRNA response elements (MRE) language, affects an RNA molecule containing MREs. Most miRNA transcripts hold numerous MREs, and these miRNAs target various mRNA transcripts downstream. As a result, a network of lncRNA/miRNA/mRNA interaction (ceRNETs) is formed which affects various phenotypes such as proliferation, metastasis, and invasion [18]. Demonstrating the same ceRNETs, a recent paper published by our lab in 2022 showed that lncRNA *LINC00324* enhances esophageal squamous cell carcinoma (ESCC) progression via the miR-493-5p/MAPK1 axis [19]. The LncRNA of our interest, *ZFAS1*, also harbors multiple miRNA binding sites, thereby regulating its target by sponging, which correlates with different disease conditions [20]. Recent studies have reported *ZFAS1*/miR-144/EZH2 axis to play a crucial role in colorectal carcinoma [21]. Similarly, studies have also reported that miR-497-5p/HMGA2, miR-200b-3p/Wnt and miR-892b/LPAR1 axis regulate malignant progression in pancreatic [12] gastric [22] and nasopharyngeal carcinoma [23] respectively. Additionally, in osteosarcoma, *ZFAS1*/miR-135a axis regulates growth and metastasis [24].

In this study, we have discussed the mechanistic and regulatory roles of *ZFAS1* in modulating the hallmarks of cancer and cellular phenotypes, including proliferation, invasion, epithelial-to-mesenchymal transition (EMT) and metastasis through sponging. *ZFAS1* sponges several miRNAs such as miR-484 [25], miR-497-5p [12], miR-892b [26], miR-10a [27], and miR-150-5p [28], whose binding sequences, as well as *p*-value and *R-*value, are listed in Table S1. We have also highlighted different signaling pathways, including molecules such as STAT3, SKA1, LPAR1 and Wnt β-catenin and their involvement in cancer development through the *ZFAS1*/miRNAs/mRNAs axis. The regulatory functions of the *ZFAS1*/miRNA/mRNAs axis might provide a prognostic and therapeutic biomarker for cancers. The in-silico analysis, including Gene Ontology (GO) and miRNA target prediction using starBase v2.0 (https://starbase.sysu.edu.cn/, accessed 10 March 2024), RNAInter v4.0 (http://www.rna-society.org/rnainter, accessed 10 March 2024) and RAID v2.0 (https://www.rna-society.org/raid/, accessed 10 March 2024) database also establish the role of *ZFAS1* as an important therapeutic biomarker. Additionally, the survival analysis of *ZFAS1* in multiple cancers illustrates its significance in patient survival. Furthermore, the Pearson correlation coefficient shows that *ZFAS1* interacts with multiple miRNAs such as miR-497-5p, miR-150-5p, miR-124-3p, miR-589-5p, and miR-7-5p and is dysregulated in various cancers.

## Methodology

### Search strategy

**Figure 2:**
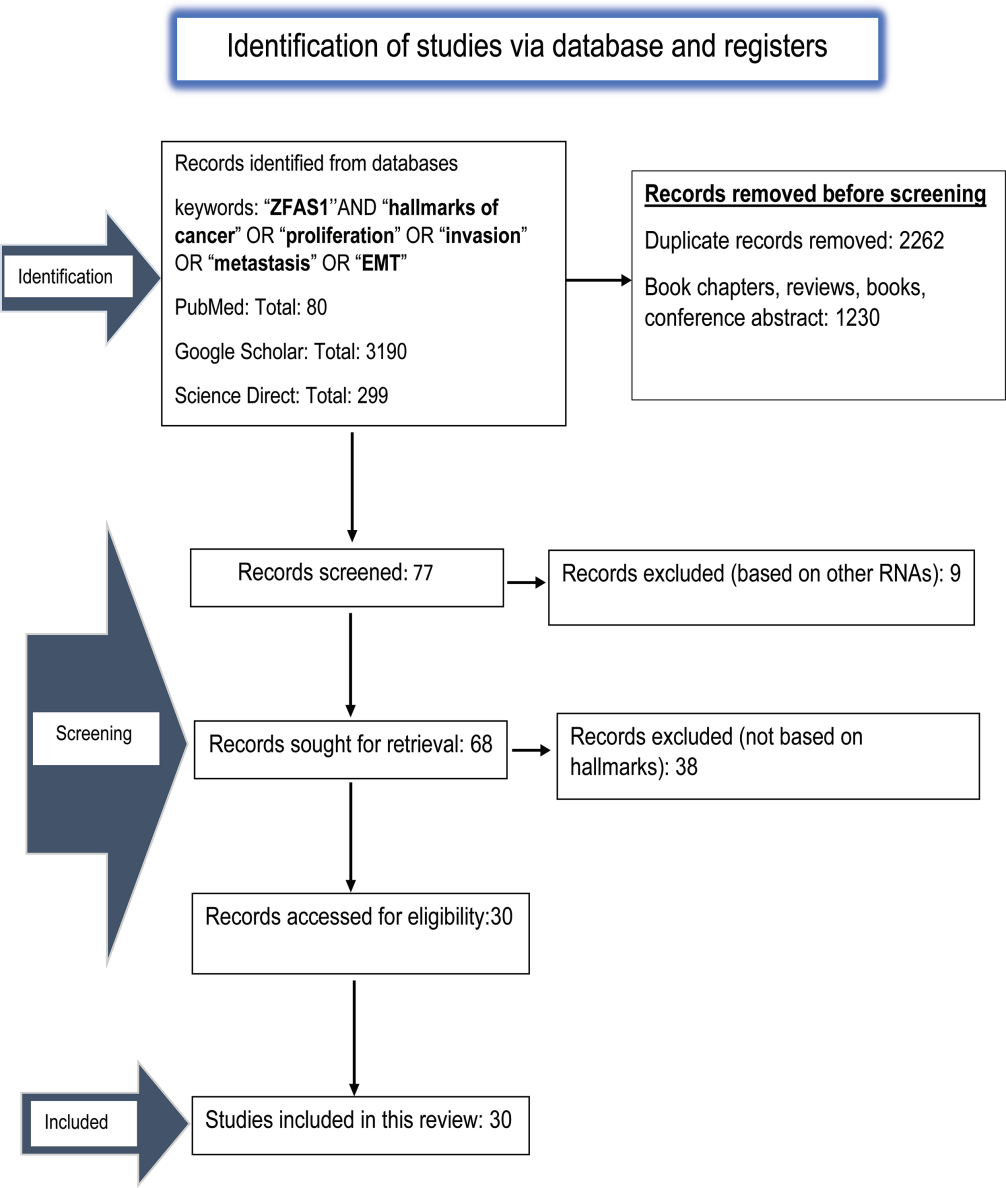
The PRISMA flow diagram depicting the process of literature search and study selection done using PubMed, Google Scholar and ScienceDirect related to *ZFAS1*/miRNA/mRNA axis and hallmarks of cancer. A total of 30 relevant articles are included in this review.

For this study, we used PubMed, Google Scholar and ScienceDirect to retrieve and assess the available literature. Two authors (S.M. and R.K.S.) independently performed the literature search and routinely discussed with an experienced researcher in consultation (A.J.) We have mentioned the screened result in Fig. 2. Relevant literature using keywords such as (“*ZFAS1”*) AND (“hallmarks of cancer”) OR (“proliferation”) OR (“metastasis”) OR (“invasion”) OR (“EMT”) were retrieved. We acquired 3190 papers from Google Scholar, 80 from PubMed, and 299 from Science Direct.

### Inclusion and exclusion criteria

Only studies correlating with *ZFAS1* and the patients’ miRNAs/mRNAs axis, cancer markers, and clinicopathological characteristics were considered. Studies that included patients’ whole blood plasma, serum, tissue samples, and experimental results were also included. Through in-depth analysis, we removed 2262 duplicate data and 1230 papers, including book chapters, reviews, books, and conference abstracts since they were not related directly to *ZFAS1*/miRNA/mRNA axis, and hallmarks of cancer. On 10 November 2023, all results were revised once more. The obtained results were screened based on the scope of this article. As a result, we got thirty articles related to the *ZFAS1*/miRNA/mRNA axis associated with different hallmarks of cancer and, therefore, eligible to be included in the study.

### Implications of ZFAS1/miRNA axis on hallmarks of cancer

A normal human cell acquires the functional abilities necessary to develop malignant tumors when it changes from a normal to a neoplastic growth state. Robert Weinberg and Douglas Hanahan illustrated this point in their 2011 paper by proposing a set of cellular phenotypic similarities that connect all cancer types. Among the functional characteristics of these malignant tumor cells are invasion and metastasis, resisting cell death, and proliferative signaling [29]. Given the fact that there are specific morphological and physiological characteristics of a tumor cell, in the sections below, we have discussed the influence of the *ZFAS1*/miRNA axis in proliferation, metastasis, EMT and invasion and the mechanisms governed by this axis in the pathogenesis of cancer.

### Unveiling the significance of the ZFAS1/miRNA axis in tumor proliferation

Growth and proliferation are crucial components of carcinogenesis, where different signal transduction pathways, including Wnt, Notch, and phosphatidyl inositol 3-kinase (PI3K/AKT), promote the division and proliferation [30]. Cell proliferation also strongly correlates with cell cycle regulation, coordinated by cyclin-dependent kinases and cyclins such as D1 and E [31]. Recent research indicates that many lncRNAs implicated in controlling cell proliferation. For instance, the DIANA bioinformatics tool affirmed that miR-484 has complementary binding sites for 3′UTR of *ZFAS1*, as listed in Table S1.

**Figure 3:**
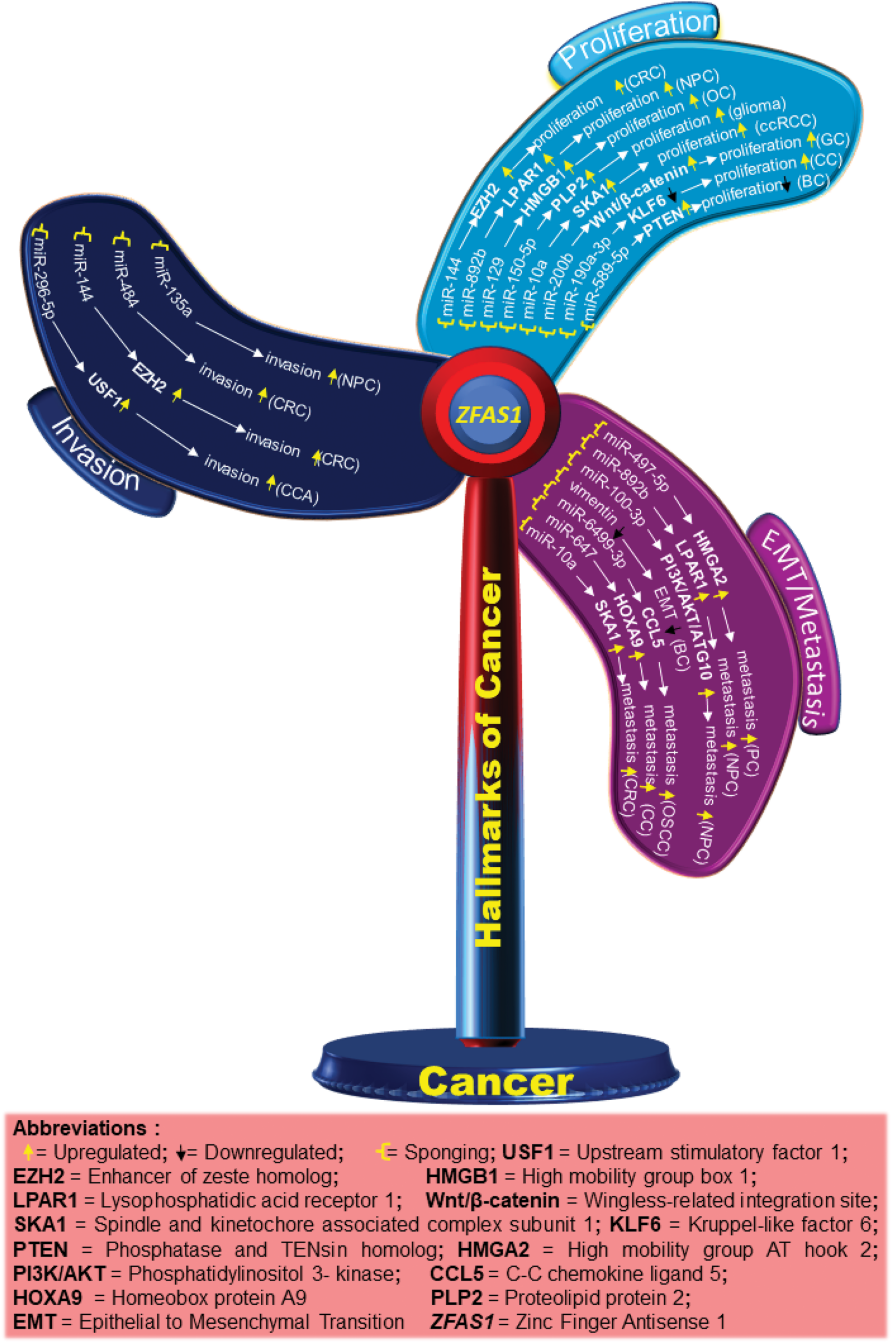
Schematic diagram showing different molecules and pathways associated with *ZFAS1* and hallmarks of cancer. The upregulated and downregulated molecules/pathways are shown by up and down arrows, respectively.

In a study, utilizing cell counting kit-8 (CCK8) it was shown that in SW480 and HT-29 colorectal cancer (CRC) cell lines, *ZFAS1* knockdown significantly inhibits cell viability and proliferation. Using luciferase reporter assay, *ZFAS1*’s interaction with miR-484 was determined (Table S1), with miR-484 mimics considerably lowering *ZFAS1’s* luciferase activity. Likewise, MTT assay analysis demonstrates that miR-484 inhibitor reverses *ZFAS1’s* suppression of SW480 cell proliferation [26]. In an independent study on colorectal cancer, *ZFAS1* has been shown to bind to Ago2, a part of the RNA-induced silencing complex (RISC) that is involved in miRNA-mediated regulation of mRNA expression. Luciferase assay revealed that *ZFAS1* binds directly to miR-144, as shown in Table S1, and *ZFAS1* activity decreased after transfection with miR-144 mimic. However, the repression of luciferase activity decreased for mutated *ZFAS1*. miR-144 was also shown to downregulate enhancer of zeste homolog 2 (EZH2) expression in LOVO and SW620 colorectal cancer cells. Here, *ZFAS1* sponges miR-144 and upregulates the expression of EZH2, leading to enhanced cell proliferation in the SW620 and LOVO cells as depicted in Fig. 3. The relationship between miR-7-5p and *ZFAS1* in CRC was confirmed by another group using luciferase assay, where overexpressed miR-7-5p reduces *ZFAS1’s* luciferase activity while *ZFAS1* acting as an oncogene, decoys miR-7-5p and increases proliferation [21]. Furthermore, rescue experiments revealed that miR-7-5p inhibitor reverses the growth inhibitory effect of *ZFAS1* knockdown in SW480 and H29 CRC cell lines [20].

In another study in CRC, *ZFAS1* downregulation inhibited the proliferation of the DLD-1 and HCT116+/+ CRC cell lines where si-*ZFAS1* arrested the cell at the G1 stage and decreased cell proliferation [14].

**Figure 4:**
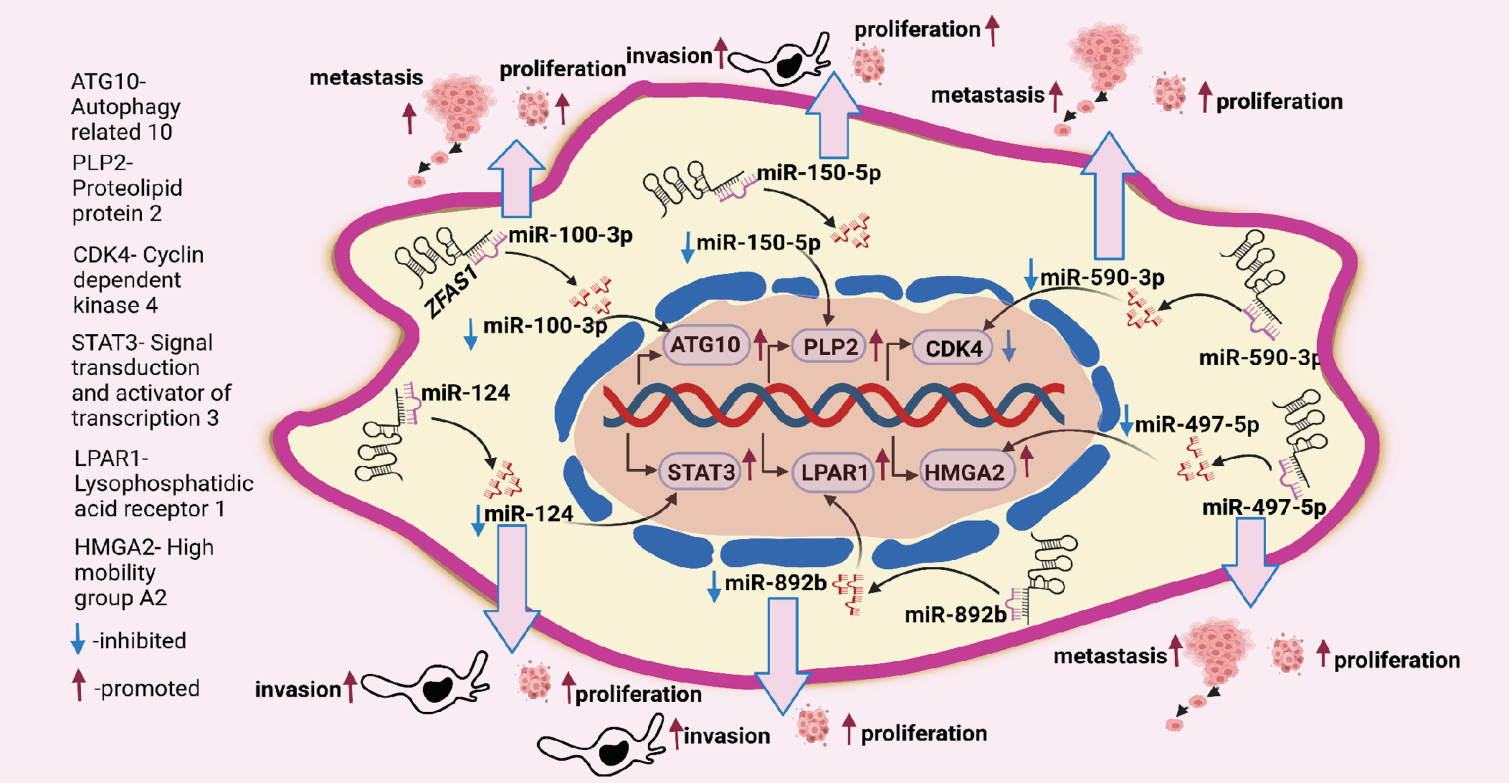
*ZFAS1* regulates cancer cell proliferation, metastasis, and invasion through sponging miRNAs. *ZFAS1* sponges miR-100-3p, miR-150-5p, miR-590-3p, miR-497-5p, miR-892b and miR-124 (Figure made in BioRender.com, accessed 10 March 2024).

CDK4/cyclin is a crucial protein for the G1/S transition during the cell cycle. *ZFAS1* silencing decreases the CDK4 levels reducing proliferation. Also, qRT-PCR analysis of 3 endometrial carcinoma (EC) cell lines, HEC-1B, Ishikawa and RL95-2 indicated higher levels of *ZFAS1* compared to human endometrial endothelial cells (hEEC). For the same, CCK8 assay analysis also reported a decrease in proliferation after *ZFAS1* knockdown in Ishikawa and RL95-2 cell lines [11]. In a different study on nasopharyngeal carcinoma, it was found that *ZFAS1* sponges miR-892b (Table S1) and upregulates the expression of LPAR1, also called endothelial differentiation gene 2 (EDG2), as shown in Figs. 3 and 4. LPAR contains fragments having an affinity for two sites of miR-892b, site 1 (nucleotide region from 440 to 886) and site 2 (nucleotide region from 1137 to 1609). Here, *ZFAS1* sponges miR-892b and inhibits the potential binding of miR-892b with LPAR1 ultimately enhancing proliferation (Fig. 3) [26].

In ovarian granulosa cells, Zhu et al. ascertained that downregulated *ZFAS1* suppresses miR-129 and upregulates the expression of high mobility group box 1 protein (HMGB1), increasing proliferation as demonstrated in Fig. 4. However, a detailed study of the *ZFAS1*/miR-129/HMGB1 axis still needs to be done. The lining of the proximal convoluted tubules is where clear renal cell carcinoma (ccRCC) starts [32]. In a study evaluating ccRCC *ZFAS1* and miR-10a are shown to have complementary binding sequences through the DIANA bioinformatics tool as mentioned in Table S1. Here, *ZFAS1* sponges miR-10a and inhibits the potential downregulation of SKA1 by miR-10a, ultimately increasing proliferation (Fig. 3). Further, SKA1 knockdown decreased proliferation in ACHN cells, indicating the direct correlation between *ZFAS1* and SKA1 [27]. In a separate study on breast cancer, the authors found that reduced *ZFAS1* expression, along with increased miR-589 expression in Michigan Cancer Foundation (MCF-7), T47D, BT-549, and MDA-MB-435 breast cancer cell lines, resulted in enhanced proliferation of the cancer cells. Furthermore, the study showed that *ZFAS1* silencing upregulates PTEN and deactivates the Phosphatidylinositol 3-Kinase (PI3K/AKT) pathway by dephosphorylating PIP3 through its sponging mechanism with miR-589 (Table S1), (Fig. 3) [33]. Similarly, upregulated *ZFAS1* enhances apoptosis by arresting the MCF-7 BC cells at the G0/G1 stage, thereby decreasing proliferation, whereas downregulated *ZFAS1* has been reported to increase proliferation in invasive ductal breast carcinoma [34].

The most prevalent form of primary liver cancer, Hepatocellular Carcinoma (HCC), develops in hepatocytes, the primary class of liver cells. In a recent study on HCC, Luo B et al. (2024) reported significant upregulation of *ZFAS1* in the tissues and cells of HCC [5]. Further knockdown of *ZFAS1* impedes the growth and movement of cells. Additionally, the knockdown of *ZFAS1* also leads to the inhibition of cell migration and proliferation [5]. Moreover, tumor development and lung metastasis decreased in mice models by *ZFAS1* knockdown [5].

The authors also showed that lncRNA ZFAS1 influences 5-aminoimidazole-4-carboxamide ribonucleotide formyl transferase/IMP cyclohydrolase (ATIC) transcription and enhances the proliferation and migration of HCC cells via the PI3K/AKT pathway. ATIC is a bifunctional protein that catalyzes the last two steps of the *de novo* purine biosynthesis. Thus, authors concluded that lncRNA ZFAS1-ATIC axis has potential as a diagnostic biomarker and therapeutic target for HCC [5].

Gastric cancer (GC) or stomach cancer is one of East Asia’s most frequent gastrointestinal malignancies [35]. Differential gene expression and chromosomal aberration are some factors in gastric cancer pathogenesis [35,36]. A study on GC reported the expression of *ZFAS1* in gastric cancer cells infected with miR-200b mimics to be significantly higher compared to NC mimics transfected cells. Gastric cancer cell lines, BGC 824 and SGC 7901, transfected with miR-200b, showed an increased expression of miR-200b, whereas cell proliferation decreased in the SGC 7901 GC cell line. The flow cytometry results revealed that overexpressed miR-200b restricts the cell cycle at the G2 phase. Moreover, through luciferase assay, the binding sites of *ZFAS1* and miR-200b and miR-200b and Wnt/β-catenin were discovered (Table S1). Here, overexpressed *ZFAS1* suppresses the expression of miR-200b in BGC 823 and SGC 7901 cell lines leading to increased Wnt/β-catenin pathway and proliferation in GC, as illustrated in Fig. 3 [22].

In nasopharyngeal carcinoma, *ZFAS1* knockdown disrupts the PI3K/AKT pathway, decreasing phosphorylated AKT (p-AKT) levels. However, no significant changes occur in total AKT levels, and treatment with 740Y-P restores the level of inhibition in cell proliferation by *ZFAS1*, where 740Y-P is a PI3K activator and plays a crucial role in PI3K/AKT signaling pathway [16]. Cell apoptosis assay of CNE2 NPC cells with si-*ZFAS1* showed many cells in the G0/G1 phase reducing the number of cells in the S phase, thus inhibiting cell proliferation by altered cell cycle progression. The authors, through western blot, analyzed that si-*ZFAS1* in CNE-2 NPC cells increased the pro-apoptotic protein p21 and p53 levels and decreased the level of anti-apoptotic protein B-cell lymphoma 2 (Bcl-2), ultimately inducing apoptosis in NPC establishing *ZFAS1* to be anti-apoptotic and an enhancer of proliferation [16]. In another study for tumor progression in temozolomide-resistant glioma cell lines U251 and U87, the authors discovered through luciferase assay and RNA pull-down assays, that *ZFAS1* sponges miR-150-5p which prevents the binding of miR-150-5p with proteolipid protein 2 (PLP2) resulting in an enhanced proliferation as shown in Fig. 3, where PLP2 is an aggressive tumor promoter, and miR-150-5p binding with PLP2 reduces proliferation [28].

Besides this, *ZFAS1* has been reported to sponge miR-329 and suppress proliferation in Bladder Cancer BLCA [37]. Cervical cancer is the cancer of the lower part uterus and cervix cells where a strong negative correlation between *ZFAS1* and miR-190a-3p has been reported. Bioinformatic tools predicted that *ZFAS1* sponges miR-190a-3p inhibiting kruppel-like factor 6 (KLF6), a tumor suppressor gene, which ultimately increases proliferation (Fig. 3, Table S1) [7].

In an independent study on osteosarcoma, *ZFAS1* knockdown reduces the proliferation of U2OS cells, the colony formation assay reveals a decrease in the number of colonies, and a strong Ki-67 signal is visible in normal cells by immunofluorescence labeling, however, *ZFAS1* knockdown U2OS cells had a remarkably weak Ki-67 signal indicating that *ZFAS1* enhances cell proliferation in osteosarcoma [14].

These mixed results support the conclusion that *ZFAS1* dysregulation affects proliferation in various cancers such as esophageal squamous cell carcinoma, gastric, breast, colorectal, hepatocellular carcinoma, cervical and nasopharyngeal carcinoma. Thus, *ZFAS1* may be used as a potential biomarker and therapeutic target in cancer treatment.

### EMT-metastasis and ZFAS1/miRNA axis an intricate path in tumorigenesis

A neoplasm or tumor is the offspring of a cell that can grow uncontrollably. Due to their unchecked growth, these benign tumors that were previously non-invasive eventually turn into malignant ones that invade blood and lymphatic vessels at random. They are carried further to distant locations where they proliferate [31]. There are defined stages of metastasis such as intravasation, transport, extravasation, colonization, and formation of micro-metastases [38]. Invasion and metastasis account for 90% of the mortality in cancer. At the time of death, most tumor cells are in the metastases [33]. Independent studies in the past decade reported abnormally expressed lncRNAs to significantly cause human tumor development and metastasis.

Recent research reports instances of metastasis are associated with epithelial-to-mesenchymal transition (EMT). EMT is a phenomenon in which the epithelial cells change cellular morphology and gain mesenchymal phenotypes under the influence of specific signaling pathways. During the transition from epithelial-to-mesenchymal properties, there is a decrease in the epithelial markers such as epithelial cell adhesion molecule (EpCAM), E-cadherin, and cadherin1 (CDH1) and an increased expression of the mesenchymal markers CDH2, Vimentin, Zinc finger E-box binding proteins (ZEB1), Snail and MMP14. This results in a loss of epithelial characters which leads to an enhancement in cell motility and invasion of nearby tissues. Multiple studies in the past decade have reported the influence of *ZFAS1* in EMT.

For instance, in pancreatic cancer, which typically begins in the cells that line the pancreatic ducts and make the hormones and digestive enzymes in the pancreas miR-497-5p acts as a tumor suppressor by regulating high mobility group A2 protein (HMGA2). *ZFAS1* sponges miR-497-5p (Table S1) thus preventing the potential binding of HMGA2 with the 3′UTR of miR-497-5p, thereby increasing the expression of HMGA2 and ultimately increasing metastasis as shown in Figs. 3 and 4 [12]. Similarly, Rao et al. studied the biological role of *ZFAS1* in pancreatic cancer by transfecting the (siRNA) of *ZFAS1* in the BxPC-3 human pancreatic adenocarcinoma cell line and SW1990 PC cell line. The results showed a decrease in metastasis, indicating that *ZFAS1* increases metastasis in pancreatic cancer [12]. Using si-*ZFAS1*, Sun et al. assessed the expression of CDK4, cyclin D1, and N-cadherin and validated the role of *ZFAS1* in EMT in EC cells. A decrease in the protein expression of CDK4, cyclin D1, and N-Cadherin, whereas an increase in the expression of E-cadherin was observed in the EC cells [11]. Similarly, in the breast cancer cell line, MDA-MB-231, western blotting results after *ZFAS1* overexpression show a decrease in mesenchymal marker Vimentin as mentioned in Fig. 3 [34].

In a study on nasopharyngeal carcinoma, by sponging miR-892b and upregulating the expression of LPAR1, *ZFAS1* enhances metastasis (Fig. 3) [26]. Similarly, the association of *ZFAS1* with the EMT-related genes significantly affects the EMT process in SGC7901 GC cells, where the western blot study reported a decrease in Vimentin and N-cadherin and an increase in the E-cadherin level in SGC7901 GC cells with si-*ZFAS1* [16]. A 2017 study reports that *ZFAS1* sponges the 3′UTR regions of miR-48 and increases metastasis in osteosarcoma (Table S1) [39]. In contrast to control cells, U2OS osteosarcoma cells with *ZFAS1* knockdown exhibit slower wound healing. In addition, the transwell assay reports a decrease in migration of U2OS cells with *ZFAS1* knockdown [13]. Furthermore, *ZFAS1* sponges miR-6499-3p in oral squamous cell carcinoma (OSCC) (Table S1). Here, miR-6499-3p negatively correlates with chemokine (C-C motif) ligand 5 (CCL5), an aggressive tumor promoter protein that significantly recruits leucocytes at the inflammation sites and initiates the formation of blood vessels. Silencing of miR-6499-3p by *ZFAS1* leads to increased CCL5 levels, thereby increasing metastasis in CAL-27 and TSCCA OSCC cells, as illustrated in Fig. 3. Also, scratch assay results show increased metastasis in CAL-27 and TSCCA OSCC cell lines with overexpressed *ZFAS1* [6]. Additionally, downregulation of *ZFAS1* decreases metastasis via the *ZFAS1*/miR-892b/LPAR1 pathway in nasopharyngeal carcinoma (Fig. 4) where *ZFAS1* sponges miR-892b increasing LPAR1 levels, thus, enhancing metastasis [26]. A biological process called autophagy involves cells breaking down and recycling parts that are not functional. For the same, acting as a ceRNA, *ZFAS1* sponges the 3' region of miR-100-3p (Table S1) and affects the level of autophagy by regulating the PI3K/AKT pathway in nasopharyngeal carcinoma. Western blot detection found that miR-100-3p inhibits the PI3K/AKT pathway downstream. Hence, silencing of miR-100-3p activates the PI3K/AKT pathway increasing the expression of ATG10 (Fig. 3), where ATG10 is an essential enzyme for autophagosome formation which carries out autophagy. Increased ATG10 leads to dysregulated autophagy and increased metastasis as shown in (Fig. 4) [23]. A different study reports that the *ZFAS1*/miR-647/HOXA9 axis regulates cervical cancer metastasis. The HOX gene family includes the (Homeobox protein A9) HOXA9, which has a DNA-binding homeodomain and is essential for hematopoiesis regulation. *ZFAS1* sponges miR-647 and, correspondingly, miR-647 overexpression reduces the luciferase activity of HOXA9, leading to a decrease in metastasis (Fig. 3) [40]. Another independent study describes the *ZFAS1*/miRNA/mRNA crosstalk in clear cell renal cell carcinoma, where results from the luciferase assay showed that *ZFAS1* has complementary binding sites for the 3'UTR of miR-10a (Table S1) and miR-10a is a tumor suppressor. The nucleotide ranges from 789 to 801 of *ZFAS1* sponges miR-10a, inhibiting the downstream targeting of SKA1 by miR-10a. Here, sponging of miR-10a increases SKA1 levels leading to an increase in metastasis as shown in Fig. 3 [27]. Moreover, qRT-PCR analysis shows that the knockdown of *ZFAS1* expression by short hairpin RNA1 (shRNA1) and short hairpin RNA 2 (shRNA2) leads to metastasis suppression [27].

In a study on HCC, *ZFAS1* decreases the tumor suppressive function of miR-150 through sponging, inhibiting the downregulation of MMP16, ZEB1, and MMP14 by miR-150, eventually increasing metastasis [35,41]. An Independent study on gastric cancer shows that the knockdown of *ZFAS1* leads to a significant decrease in the expression of MMP9, MMP2, ZEB1, N-cadherin, Integrin β1, and Snail while upregulating E-cadherin leading to an increase in metastasis [42].

### Explicating invasion and ZFAS1/miRNA affinities

In invasion, malignant tumor cells separate from the primary tumor mass due to a loss of cell-cell adhesion capacity. Invasion to nearby cells is also aided by modifications in the cell-matrix interaction. The tumor cells distort their cytoplasm and squeeze through the small openings to invade the nearby cells [38]. Independent reports on various lncRNAs in the past decade report the role of *ZFAS1* as an essential marker for invasion in tumor cells. For instance, in a typical head and neck malignancy of nasopharyngeal carcinoma, when CNE1 and HONE1 NPC cell lines are transfected with si-*ZFAS1*, it showed a decrease in invasive abilities, while overexpression of miR-135a resulted in the suppressed invasion. Luciferase assay and starBase bioinformatic tool showed potential binding sites between *ZFAS1* and miR-135a as listed in Table S1 [43].

A different study on cholangiocarcinoma (CCA) luciferase assay reports that *ZFAS1* sponge the 3'UTR of miR-296-5p inhibiting the downstream binding of miR-296-5p with the 5' region of upstream stimulatory factor 1 (USF1), resulting in enhanced invasion (Table S1, Fig. 3), [9]. Similarly, in a study on colorectal cancer, si-*ZFAS1* significantly reduced invasion in SW480 and HT-29 CRC cell lines. Here, *ZFAS1* was shown to sponge miR-484 as listed in Table S1, further increasing the invasion in SW480 and HT-29 CRC cell lines as shown in Fig. 3. qRT-PCR results further confirmed the negative correlation between miR-484 to *ZFAS1* [26]. Furthermore, *ZFAS1* regulates miR-144 gene expression by sponging and titrating off its binding with protein-coding messengers in CRC (Table S1). miR-144, generally downregulated in various tumors, functions as a tumor suppressor and is downregulated in colorectal cancer. miR-144 is sponged by *ZFAS1*, preventing the binding of miR-144 with 3'UTR EZH2, leading to an increase in the concentration of EZH2 in cells and tissues which ultimately increases invasion (Fig. 3) [21].

Another study demonstrates a significant decrease in the invasive potential of the ACHN and Caki-1 ccRCC cells using transwell migration and invasion assays on the knocked-down *ZFAS1*. Here, *ZFAS1* was shown to sponge miR-10a and upregulate the expression of SKA1 protein thereby increasing invasion (Table S1) [27]. Furthermore, si-*ZFAS1* decreases the invasion by inhibiting EMT through the lowering of ZEB1, N-cadherin, MMP2, MMP9, and Integrin expressions, while upregulating E-cadherin levels in glioblastoma [10]. Similar research on gliomas used western blot to assess the expression of EMT markers MMP9, MMP2, E-cadherin, Integrin1, ZEB1, Twist, and Snail and discovered a correlation with invasion. Additionally, the result of the western blot suggested that the expression of *ZFAS1* was correlated with an increase in the mesenchymal markers N-cadherin, Integrinβ1, ZEB1, and Twist. Corresponding transwell assay on U87 and U251 glioma cells showed an increase in invasion compared to the normal tissues [10].

*ZFAS1* also inhibits breast cancer cell invasion by targeting miR-589 and suppressing the PI3K/AKT signal pathway (Table S1) [33]. Similarly, *ZFAS1* inhibits invasion by sponging miR-7-5p in colorectal cancer (Table S1) [20].

The above findings suggest that *ZFAS1* is involved in the etiology of multiple cancer invasions via pathways such as miR-10a/SKA1 pathway and PI3K/AKT. Its dysregulation leads to invasion in various types of cancers. Thus, it might serve as a potential biomarker for cancer prognosis and treatment.

### In-silico target analysis of ZFAS1

**Figure 5:**
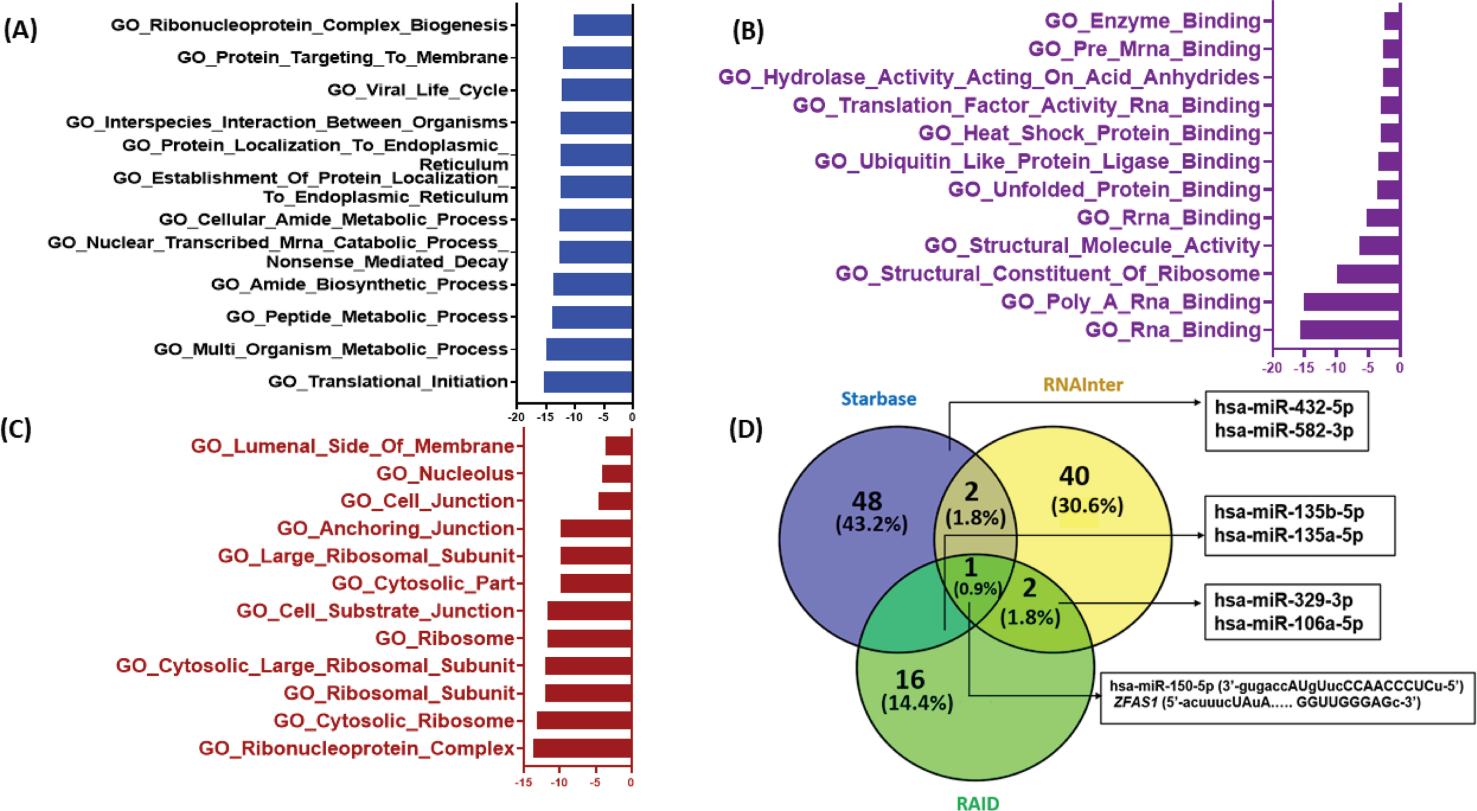
In-silico target prediction of lncRNA *ZFAS1* using online databases starBase v2.0, RNAInter v4.0 and RAID v2.0. (A) GO Biological processes (B) GO Molecular processes (C) GO Cellular compartment (D) Venn diagram for miRNA targets of *ZFAS1* made using Venny 2.0.

To study the possible molecular targets and pathways associated with *ZFAS1* the Gene Ontology (GO) pathway analysis was done (Fig. 5A–C). The GO helps to determine gene/gene product in detail, considering important aspects such as its biological process, molecular function, and cellular location. The *ZFAS1* sequence was searched on starBase v2.0 for biological, molecular, and cellular activities. We have demonstrated the results using GraphPad Prism v8.0. The processes and pathways are shown on the *Y*-axis, while the –log_10_ (false discovery rate), i.e., –log_10_ (FDR), is shown on the *X*-axis. These crucial values suggest a pivotal role of *ZFAS1* in translation initiation, as depicted in GO biological processes (Fig. 5A). Furthermore, *ZFAS1* is vital in RNA binding, as illustrated in GO molecular processes Fig. 5B. Additionally, the GO cellular compartment shows the involvement of *ZFAS1* in complex ribonucleoprotein formation (Fig. 5C). GO results may help in the future development of biomarkers linked with *ZFAS1*.

To find the common miRNA targets of *ZFAS1*, starBase v2.0, RNAInter v4.0, and RAID v2.0 databases were used. A Venn diagram depicting these miRNA targets of *ZFAS1* is shown in Fig. 5D. Interestingly, we discovered 7 common miRNA targets including hsa-miR-150-5p, hsa-miR-582-3p, hsa-miR-432-5p, has-miR-329-3p, hsa-miR-106a-5p, miR-135b-5p, miR-135a-5p. Out of the above-mentioned seven miRNAs, hsa-miR-150-5p was discovered as the common miRNA target after the analysis of the three databases mentioned above. hsa-miR-150-5p is found on chromosome 19 and has a negative correlation with *ZFAS1*. The binding of *ZFAS1* and miR-150-5p modifies cancer-related phenotypes. For instance, *ZFAS1* sponges hsa-miR-150-5p (*R* = −0.346, *p*-value = 3.27e−16) and prevents the binding of miR-150-5p and PLP2, thereby, enhancing proliferation in glioma [28]. Also, hsa-miR-150-5p suppresses tumor progression in colorectal cancer by targeting vascular endothelial growth factor A (VEGFA) [44]. Additionally, starBase v2.0 and RNAInter v4.0 analysis showed hsa-miR-432-5p and hsa-miR-582-3p as *ZFAS1*’s miRNA targets. hsa-miR-432-5p and hsa-miR-582-3p are found on chromosome 14 and chromosome 5 respectively. Studies report hsa-miR-432-5p to inhibit cell migration and invasion in colorectal cancer by targeting C-X-C motif chemokine ligand 5 (CXCL5) [45] whereas hsa-miR-582-5p suppresses ovarian cancer progression by targeting the AKT/mTOR pathway [46]. Similarly, hsa-miR-135b-5p and hsa-miR-135a-5p were discovered as *ZFAS1*’s predicted miRNA target by starBase v2.0 and RAID v2.0 where hsa-miR-135b-5p is found on chromosome 1 [47]. Inhibition of hsa-miR-135b-5p expression reduces proliferation, migration, and invasion in colorectal cancer [48]. While hsa-miR-135a-5p binds to *ZFAS1* and regulates tumor growth and metastasis in osteosarcoma [24] Furthermore, the binding of *ZFAS1* to hsa-miR-135a-5p leads to increased invasion in nasopharyngeal carcinoma [43]. RNAInter v4.0 and RAID v2.0 predicted hsa-miR-329-3p and hsa-miR-106a-5p as the miRNA targets of *ZFAS1*, and hsa-miR-106a-5p is present on chromosome X. In patients with cervical cancer, downregulation of hsa-miR-329-3p renders a worse prognosis [49] and by targeting KLF6, the miR-106a-5p carried by tumor-derived extracellular vesicles enhances the invasion and metastasis of ovarian cancer [50]. The obtained in-silico data opens a new avenue for further wet lab validations.

### Survival analysis and prognostic value of ZFAS1

**Figure 6:**
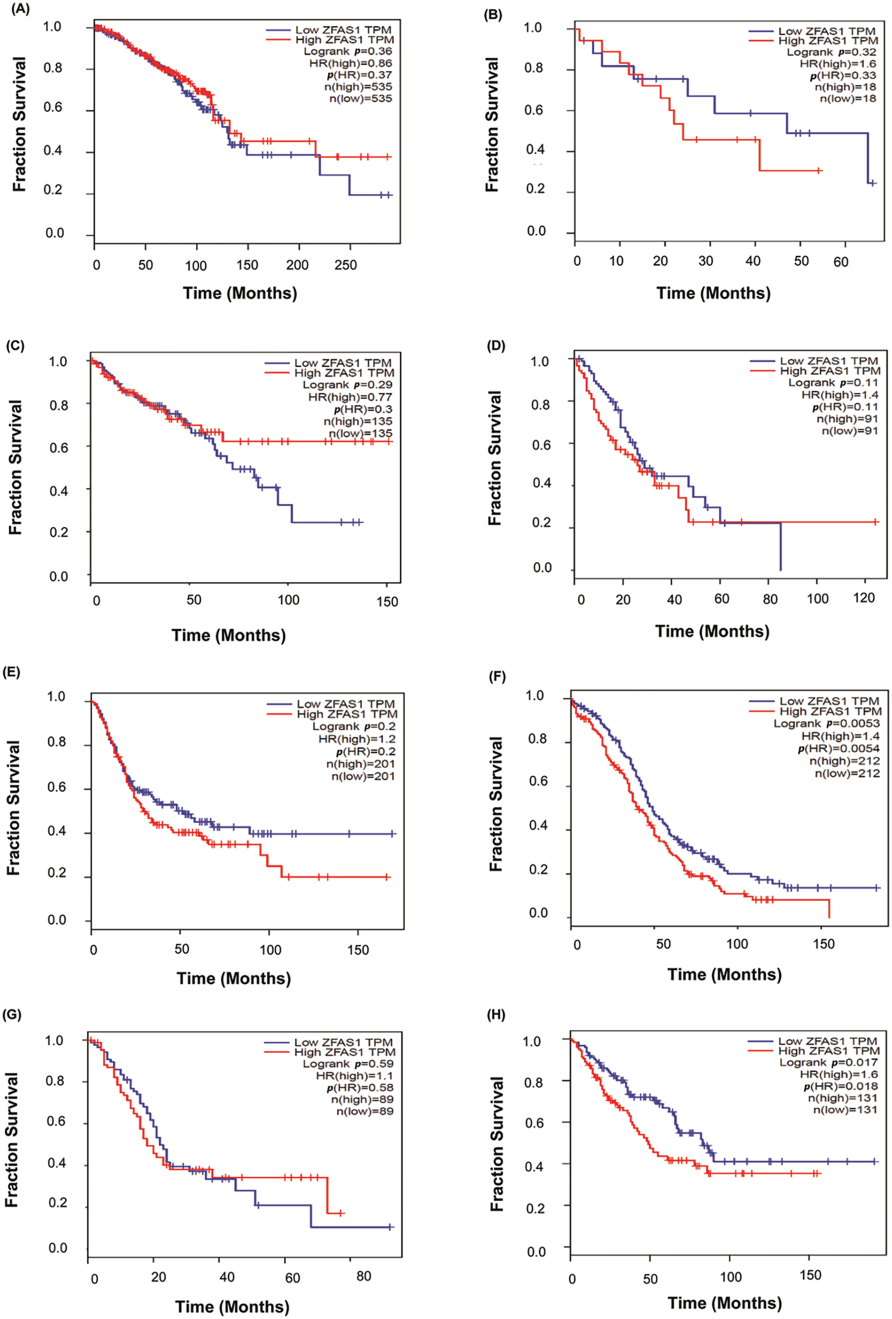
Plots of Kaplan-Meier estimate overall survival of a group of patients retrieved from TCGA database (https://ualcan.path.uab.edu/, accessed on 10 March 2024). (A) Breast cancer (BC) (B) Cholangiocarcinoma (CCA) (C) Colorectal cancer (CRC) (D) Esophageal squamous cell carcinoma (ESCC) (E) Bladder cancer (BLCA) (F) Ovarian cancer (OC) (G) Pancreatic cancer (PC) (H) Sarcoma (SARC).

Furthermore, to study the survival analysis and prognostic value of *ZFAS1* in different cancers, *ZFAS1*, and miRNA were searched as categories in the TCGA database. The patient survival duration of low *ZFAS1* is more than high *ZFAS1* in cholangiocarcinoma, esophageal squamous cell carcinoma, bladder cancer, ovarian cancer, pancreatic cancer, and sarcoma. In contrast, the patient survival of high *ZFAS1* is more than low *ZFAS1* in colorectal cancer and esophageal squamous cell carcinoma. However, patient survival duration of low and high *ZFAS1* does not significantly differ in breast cancer. Kaplan-Meier plot illustrating breast cancer (*p*-value = 0.36), cholangiocarcinoma (*p*-value = 0.32), colorectal cancer (*p*-value = 0.29), esophageal squamous cell carcinoma (*p*-value = 0.29), bladder cancer (*p*-value = 0.2), ovarian cancer (*p*-value = 0.0053), pancreatic cancer (*p*-value = 0.059), and sarcoma (*p*-value = 0.017) is as shown in Fig. 6A–H. The analysis shows significant *p*-value in ovarian cancer, pancreatic cancer, and sarcoma. However, no significant *p*-value is observed in the case of breast cancer, cholangiocarcinoma, colorectal cancer, esophageal squamous cell carcinoma, and bladder cancer.

## Conclusion and Future Perspectives

Although innovative diagnostic and prognostic methods for cancer treatment have been developed globally, an increasing number of studies establish the fact that lncRNAs play an essential role in transcriptional, post-transcriptional, translational, and normal functioning of cellular machinery, such as metastasis, cell proliferation, growth, apoptosis, and migration. An equilibrium of lncRNAs in the cellular environment is vital for the normal functioning of a cell. At the same time, their imbalance leads to the progression of various cancer types. Based on the literature discussed above, therapeutic targeting of *ZFAS1* may serve as a potential strategy for diagnosing and treating multiple cancers by mitigating STAT3, AKT, and SKA1 pathways in both tumor and non-tumorous components of tumor-microenvironment (TME). The tumor microenvironment is composed of cancer cells, endothelial cells, immunological cells (neutrophils, dendritic cells, B cells, T cells, macrophages, NK cells), cancer-associated fibroblasts, myofibroblasts, mesenchymal cells, epithelial cells, and stromal cells. Cancer cells can precisely reprogram healthy surrounding cells to favor tumor growth [51]. Exosomes are produced into TME from cancer cells containing *ZFAS1* that have the potential to control immune system function, angiogenesis, metastasis, and cancer growth and migration [52,53]. Moreover, *ZFAS1* can influence the expression of various oncogenes, transcription factors, and cellular machinery involving tumor etiology and progression, affecting cell metabolism, invasion, metastasis, proliferation, and cell resistance to death. lncRNA *ZFAS1* functioning as a ceRNA for the miRNA and subsequently upregulating or downregulating the protein synthesis has been the center of this paper. We have discussed different pathways in this paper that link the *ZFAS1*/miRNA/mRNA axis and regulate protein expression thereby altering cell phenotypes. For instance, *ZFAS1* enhances cell proliferation in gastric cancer through the Wnt/β-catenin pathway. Likewise, by regulating the SKA1 pathway, *ZFAS1* increases cell proliferation in clear-cell renal cell nasopharyngeal carcinoma through PI3K/AKT carcinoma. ZFAS1 shows enhanced metastasis in the pathway. Whereas, in the case of breast cancer, downregulation of *ZFAS1* leads to an increase in the expression of miR-589, which further causes a decrease in the level of PTEN in breast cancer cells. These interactions ultimately lead to the expression or suppression of specific proteins, affecting cells’ morphology and physiology.

**Figure 7:**
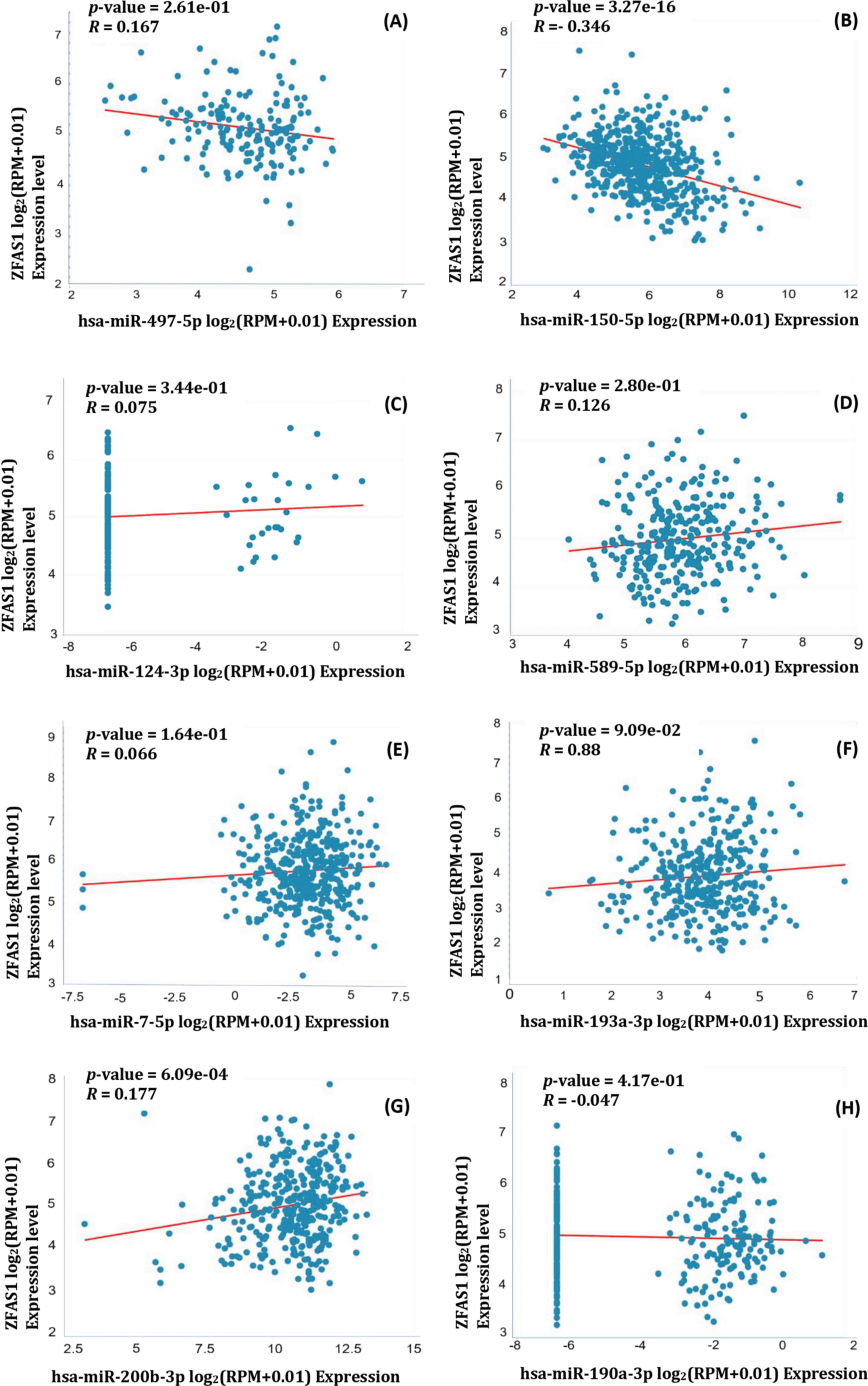
Pearson correlation coefficient of *ZFAS1* and miRNA obtained using starBase v2.0. (A) hsa-miR-497-5p (B) hsa-miR-150-5p (C) hsa-miR-124-3p (D) hsa-miR-589-5p (E) hsa-miR-7-5p (F) hsa-miR-193a-3p (G) hsa-miR-200b-3p (H) hsa-miR-190a-3p.

Additionally, from the Pan-Cancer miRNA Target Co-Expression Analysis of *ZFAS1* and different miRNAs using the starBase v2.0 (https://starbase.sysu.edu.cn/, accessed on 10 March 2024), the binding affinities were obtained. *ZFAS1* shows negative correlation with hsa-miR-497-5p (*R* = −0.167, *p*-value = 2.61e−01), and hsa-miR-150-5p (*R* = −0.346, *p*-value = 3.27e−16). Also, *ZFAS1* positively correlates with hsa-miR-124-3p (*R* = 0.075, *p*-value = 3.44e−01), hsa-miR-589-5p (*R* = 0.126, *p*-value = 2.80e−01), hsa-miR-7-5p (*R* = 0.066, *p*-value = 1.64e−01), hsa-miR-193a-3p (*R* = 0.088, *p*-value = 9.09e−02), hsa-miR-200b-3p (*R* = 0.177, *p*-value = 6.09e−04), hsa-miR-190a-3p (*R* = −0.047, *p*-value, *R* = 4.17e−01) (Fig. 7A–H). The negative correlation between *ZFAS1* and miRNAs indicates that both work antagonistically. Also, a positive correlation between *ZFAS1* and miRNA shows that both work synergistically. The significant *R-*value and *p*-value of hsa-miR-150-5p, along with prediction from three different databases as common miRNA targets indicate its potential role in other cancers apart from glioma. The knowledge obtained from this paper may provide the development of hsa-miR-150-5p as a crucial molecule in modulating cancer hallmarks and as a new therapeutic target and biomarker for cancer treatment.

Since *ZFAS1* dysregulation is evident in cancers, its profiling may create a paradigm in personalized medicine for future cancer-oriented interventions. It is possible to use exosomes as carriers to transport *ZFAS1* to tissues or organs by altering the isolated and purified exosomes with mimics or inhibitors and target molecules loaded on their surface [54]. Thus, we propose *ZFAS1* as a prominent prognostic and therapeutic biomarker in cancer-directed interventions. Its targeting may foster significant advances in the prognosis and treatment of multiple cancer types. Since the *ZFAS1*/miRNAs/mRNAs regulatory axis plays a crucial role in various biological mechanisms, including cancer progression, it offers a potential avenue for therapeutic intervention in cancer treatment. Strategies to target this axis include the employment of antisense oligonucleotides (ASO) or *ZFAS1* mimics. Additionally, the use of miRNA mimics or inhibitors and gene inhibitors represents another approach to inhibit cancer growth. To enhance the cellular penetration of these RNAs, it is necessary to improve their stability against degradation by utilizing optimized chemical structures [53] lipid-based delivery vehicles, polymeric nanoparticles, and viral systems that can be utilized in similar pharmaceutical formulations to increase stability and improve the pharmacokinetic potential of the associated oligonucleotides [55–58]. It is also possible to use exosomes as carriers to transport *ZFAS1* mimics or inhibitors to tissues or organs by targeting specific proteins loaded on their surface [53,54]. Despite the absence of clinical trials for *ZFAS1* inhibitors or mimics in cancer therapy, several miRNAs, such as miR-10b, are undergoing clinical trials (e.g., NCT01849952 for Glioma), indicating progress in exploiting this regulatory axis for cancer treatment.

As *ZFAS1* dysregulation is evident in cancers, its profiling may create a paradigm in personalized medicine for future cancer-oriented interventions. Thus, we propose *ZFAS1* as a prominent prognostic and therapeutic biomarker in cancer-directed interventions. Its targeting may foster significant advances in the prognosis and treatment of multiple cancer types.

## Limitations to Our Study

Although several lncRNAs, including *ZFAS1*, have been reported recently, scientific knowledge regarding the effects of lncRNA on targeted therapeutics is still limited. Hence, further studies are required regarding the vital cellular signaling molecules and discrete pathways dysregulated by distinct lncRNAs in cancer. Also, the role of *ZFAS1* in several cancers has been well studied *in vitro*, but very few *in vivo* models have been investigated. Delivering lncRNA or miRNA into the cell to analyze its impact on cellular phenotypes is challenging because of the associated risks of its degradation and reduced stability after being introduced into the cell.

## Supplementary Materials







## Data Availability

Not applicable.

## Abbreviations

***ZFAS1***: Zinc Finger Antisense 1
**STAT3**: Signal Transducer and Activator of Transcription 3
**LPAR1**: Lysophosphatidic acid receptor 1
**SKA1**: Spindle and Kinetochore-associated Protein 1
**HMG**: High mobility group protein
**KLF6**: Kruppel-like factor 6

## Supplementary Materials

The supplementary material is available online at https://doi.org/10.32604/or.2024.050548.
