# Supplementary material for: The regulatory role of ZFAS1/miRNAs/mRNAs axis in cancer: a systematic review
Source: Oncol Res. 2025 Feb 28;33(3):591–604. doi: 10.32604/or.2024.050548 (PMC11915068; doi:10.32604/or.2024.050548)
Supplement: Supplementary file 1 [file OncolRes-33-50548-s001.docx]

**Supplementary Table S1**: Sponging sites between *ZFAS1*/ miRNAs and the associated cancer. The binding sequences are obtained from starBase v2.0 and DIANA. The p-values and R-values are obtained from Starbase. The bold and capital letters denote complementary binding sites.

| ***ZFAS1/*miRNA binding sites** | **Cancer** | ***p*-value** | ***R*-value** |
| --- | --- | --- | --- |
| *ZFAS1*-(‘**5**-uccaca**AGG**uu**ACUG**uauacau**AGCCUGA**-**3’**)  miR-484-(**3’**-c**UCC**cc**UGACUCGGACU**-**5’**) | CRC | 9.35e-01 | 0.006 |
| *ZFAS1-* **(5’-**ucugcugaaccaguuccacaaggu**UACUGUA**ua**-3’)**  miR-144-**(3’-**ucauguaguagau**AUGACAU-5’)** | CRC | 2.72e-01 | - 0.087 |
| *ZFAS1-***(5’...**aaguggugccaacac**GUCUUCC**c**...3’)**  miR-7-5p-**(3’**...uguuguuuagugau**CAGAAGG**u...**5’)** | CRC | 1.64e-01 | 0.066 |
| *ZFAS1*-(**5’**a**ACAGA**….u**ACAGGUA**-**3’**)  miR-10a-(**3’**g**UGUUU**aagccuag**AUGUCCAU**-**5’**) | ccRCC | 1.16e-02 | 0.199 |
| *ZFAS1-*(**5’**-aucucuacacuauu**GGCCAGUU**-**3’**)  miR-193a-3p-(**3’**-ugacccugaaacau**CCGGUCAA**-**5’**) | HCC | 9.09e-02 | 0.088 |
| *ZFAS1*-(**5’**-agaaaa**GG**a..**CA**ga**CCCT**g**T**g-**3’**)  miR-135a-(**3’**-gcggtg**CC**gag**GT**ta**GGGA**t**A**t-**5’**) | osteosarcoma | 9.07e-02 | - 0.088 |
| *ZFAS1-*(**5’**-acuuuc**UA**u**A**….**GGUUGGGAG**c-**3’**)  miR-150-5p-(**3’**-gugacc**AU**g**U**uc**CCAACCCUC**u-**5’**) | glioma | 3.27e-16 | - 0.346 |
| *ZFAS1-*(**5’-GG**a**CA**g—**ACC**ct**GTGC**t**TT**c-**3’**)  miR-124-(**3’**-**CC**—**GT**aag**TGG**cg**CACG**g**AA**t-**5’**) | ESCC | 3.4e-01 | 0.075 |
| *ZFAS1-*(**5’**-cggguguguuggaaguagaauau**AUAUAUA**c-**3’**)  miR-190a-3p-(**3’**-uccuuauacaaac**UAUAUAU**c-**5’**) | CC | 2.80e-02 | 0.126 |
| *ZFAS1-*(**5’**-gcgccucgggc**UGUGCUGCU**c-**3’**)  miR-497-5p-(**3’**-uguuugguguc**ACACGACGA**c-**5’**) | PC | 2.67e-01 | - 0.052 |
| *ZFAS1-*(**5’**-ggaaatacg...**AGTACAGG**g…**3’**)  miR-486-(**3’**-gagccccgtcgag**TCATGTCC**t-**5’**) | osteosarcoma | 6.58e-03 | 0.168 |
| *ZFAS1-*(**5**’-u**GGG**c**AAGU**uuuuc**AG**acac**UGCAGCC**Ag-**3’**)  miR-647-(**3’-**u**UCCUUCA**c**UC**acac**ACGUCGGU**g**-5’**) | CC | 9.57e-01 | - 0.003 |
| *ZFAS1*-(**5’**-uggccaaa**ACCAGGC**uuug**AUU**ga…**3’**) (654-677)  miR-200b-3p-(**3’**-aguaguaa**UGGUCCG**--uca**UAA**u-**5’**)  *ZFAS1*-(**5’**-ucugcgg**U**g**CC**c**GG**—**AGU**g**U**g…**3’**) (473-492)  miR-200b-3p-(**3’**-aguagua**A**u**GG**u**CC**g**UCA**u**A**au-**5’**) | GC | 6.09e-04 | 0.177 |
| *ZFAS1-*(**5’**-**GGGGGCCC**a-**3’**)  miR-296-5p-(**3’**-uguccuaacucc**CCCCCGGG**a-**5’**) | CCA | 4.42e-01 | 0.132 |

| *ZFAS1*-(**5’**-ctgcccgttagagc**AGCCAG**cgggt-**3’**)  miR-892b-**(3’***-*agatgggtctttcc**TCGGTC**ac***-*5’)** | NPC | - | - |
| --- | --- | --- | --- |
| *ZFAS1*- (**5’**-gagccu**GCAGA**g**G**gcc**GGUUCUC**c-**3’**)  miR-589-(**3’**gagucu**CGUCUGC**….**ACCAAGAG**u-**5’**) | BC | - | - |
| *ZFAS1-*(**5’**-acaugg**AUAG**uaagg**AAGCUU**ag-**3’**)  miR-100-3p-(**3’**-guaugga**UAUC**uaug…**UUCGAA**c-**5’**) | NPC | - | - |
| *ZFAS1-*(**5’**-gcaga**UGGGCAAG**uuuuu**CAGACACUGC**a-**3’**)  miR-6499-3p-(**3’**-c**ACCCGUUU**u**GUUUGUGACG**a-**5’**) | OSCC | - | - |
| *ZFAS1-*(**5’**…auauaagggagguucagg**AAGCCAU**u…-**3’)**  miR-135a-(**3’-**aguguauccuuauuu**UuCGGUA**u-**5’**) | NPC | - | - |
| *ZFAS1-*(**5’**-agccacgugu**ACCAG**u**GUGUGU**u-**3’**-)  miR-329-(**3’**-uuucuccaau**UGGUC**-**CACACA**a-**5’**) | BLCA | - | - |

**Abbreviations**

**CRC** = Colorectal cancer **GC** = gastric cancer **BLCA** = bladder cancer

**HCC** = hepatocellular carcinoma **BC** = breast cancer

**ESCC** = esophageal squamous cell carcinoma **NPC** = nasopharyngeal carcinoma

**CC** = cervical cancer **CCA** = cholangiocarcinoma

**PC** = pancreatic cancer **OSCC** = oral squamous cell carcinoma
